# Supplementary material for: Identification of photoperiod-induced specific miRNAs in the adrenal glands of Sunite sheep (Ovis aries)
Source: Front Vet Sci. 2022 Jul 22;9:888207. doi: 10.3389/fvets.2022.888207 (PMC9354845; doi:10.3389/fvets.2022.888207)
Supplement: Supplementary file 1 [file Data_Sheet_1.ZIP › Supplementary file/miRDeep2 file.zip!/miRDeep2 file/Novel_Structure/LP42A1/Novel_700.pdf]

Provisional ID	:	NC_019483.2_34743
Score total	:	20.7
Score for star read(s)	:	3.9
Score for read counts	:	12.9
Score for mfe	:	2.3
Score for randfold	:	1.6
Score for cons. seed	:	
Total read count	:	37
Mature read count	:	36
Loop read count	:	0
Star read count	:	1

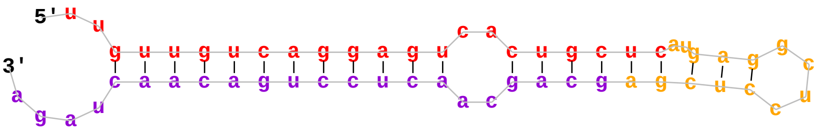

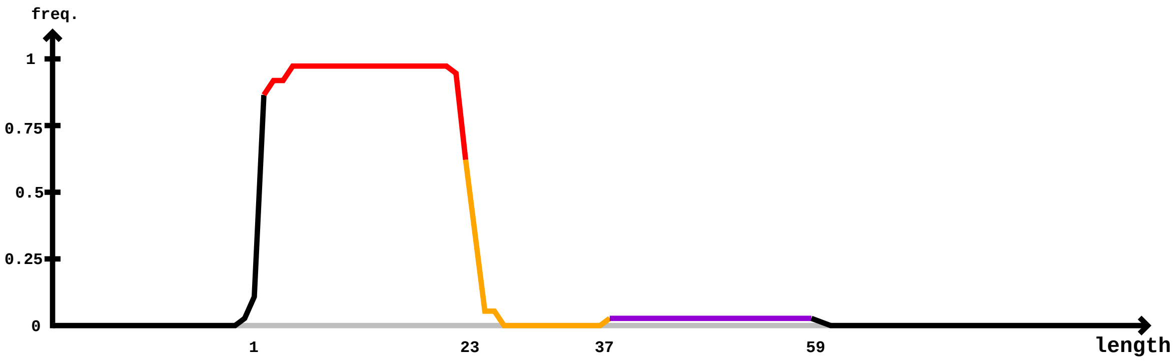


```
                         Mature                         Star
5'- gacgauguaggcucacccacuuguugucaggagucacugcucaugaggcuccucgagcagcaacuccugacaacuagaagggagggcagagcaucagcgcagccccugugga  -3'   obs
    gacgauguaggcucacccacuuguugucaggagucacugcucaugaggcuccucgagcagcaacuccugacaacuagaagggagggcagagcaucagcgcagccccugugga         exp
    ...(((((..((((.(((.((.((((((((((((..((((((..(((....)))))))))..)))))))))))).))..))).))))...)))))..(((((...)))))..   reads   mm   sample
    ..................acuuguugucaggagucacugcu.......................................................................   1       0    seq
    ...................cuuguugucaggagucacugcu.......................................................................   2       0    seq
    ...................cuuguugucaggagucacugcuca.....................................................................   1       0    seq
    ....................uuguugucaggagucacugc........................................................................   1       0    seq
    ....................uuguugucaggagucacugcu.......................................................................   9       0    seq
    ....................uuguugucaggagucacugcuc......................................................................   14      0    seq
    ....................uuguugucaggagucacugcuca.....................................................................   4       0    seq
    .....................uguugucaggagucacugcuc......................................................................   2       0    seq
    .......................uugucaggagucacugcucaug...................................................................   2       0    seq
    ........................................................gcagcaacuccugacaacuaga..................................   1       0    seq
```
